# PHF19 promotes the proliferation, migration, and chemosensitivity of glioblastoma to doxorubicin through modulation of the SIAH1/β–catenin axis

**DOI:** 10.1038/s41419-018-1082-z

**Published:** 2018-10-15

**Authors:** Qing Deng, Jianbing Hou, Liying Feng, Ailing Lv, Xiaoxue Ke, Hanghua Liang, Feng Wang, Kui Zhang, Kuijun Chen, Hongjuan Cui

**Affiliations:** 1 State Key Laboratory of Silkworm Genome Biology, The Institute of Sericulture and Systems Biology, Southwest University, 400716 Chongqing, People’s Republic of China; 2 Department 6 of the Research Institute of Surgery, State Key Laboratory of Trauma, Burns and Combined Injury, Daping Hospital, Army Medical University, 400042 Chongqing, People’s Republic of China

## Abstract

PHD finger protein 19 (PHF19), a critical component of the polycomb repressive complex 2 (PRC2), is crucial for maintaining the repressive transcriptional activity of several developmental regulatory genes and plays essential roles in various biological processes. Abnormal expression of PHF19 causes dysplasia or serious diseases, including chronic myeloid disorders and tumors. However, the biological functions and molecular mechanisms of PHF19 in glioblastoma (GBM) remain unclear. Here, we demonstrated that PHF19 expression was positively associated with GBM progression, including cell proliferation, migration, invasion, chemosensitivity, and tumorigenesis. Using XAV-939, a Wnt/β-catenin inhibitor, we found that the effects of PHF19 on GBM cells were β-catenin-dependent. We also demonstrated that PHF19 expression was positively correlated with cytoplasmic β-catenin expression. PHF19 stabilized β-catenin by inhibiting the transcription of seven in absentia homolog 1 (SIAH1), an E3 ubiquitin ligase of β-catenin, through direct binding to the SIAH1 promoter region. Taken together, our results revealed the novel PHF19-SIAH1–β-catenin axis as a potential and promising therapeutic target.

## Introduction

Glioblastoma (GBM), an astrocytoma classified as grade IV by the World Health Organization, is the most common and most aggressive form of human adult brain tumors, with an incidence of approximately 3.19/100,000 per year^1^. The aggressiveness of GBM is manifested in its invasion and destruction of normal brain parenchyma, intratumoral heterogeneity, and drug resistance^2^. Currently, GBM treatment is limited to chemotherapy, radiotherapy, and surgical resection^3^. However, the prognosis of GBM patients remains poor^2,4^, with an average survival of only 14 months^5^. Due to the high mortality and morbidity of GBM and the limited treatment regimens, development of new targeted therapy strategies is urgently needed.

Polycomb group proteins are chromatin-related gene repressors that play an important role in embryonic development, stem cell differentiation, and cell proliferation^6^. Polycomb members form protein complexes, and the most common of these complexes are polycomb repressive complex 1 (PRC1) and PRC2^7^. PRC2, a key mediator of tumor cell plasticity that is required for the adaptation of GBM cells to their microenvironment, exerts oncogenic effects in many tumor types. PHD finger protein 19 (PHF19), also named PCL3^8^, is an essential component of PRC2^9–11^ and has been proposed to modulate the enzymatic activity of PRC2. PHF19 was first identified more than 30 years ago and was shown to be essential for maintaining the normal status of many body parts during *Drosophila* development^8^. Recently, several studies have confirmed that PHF19 is upregulated in many types of cancer tissues compared with the corresponding normal tissues^12–14^. These studies suggested that PHF19 is closely related to aggressive tumor behavior and is increased in various human tumor types.

Wnt/β-catenin signaling affects important cancer functions, including invasion, cell proliferation, and transformation^10^. β-Catenin is continuously activated in a variety of tumors, including the most malignant form of glioma (GBM)^15^. High expression of β-catenin has a poor prognostic impact on GBM patients^16^. Many post-translational modifications, including phosphorylation, ubiquitination, and acetylation, are involved in regulating β-catenin function^17^. Therefore, tight regulation of β-catenin expression is required.

The regulatory mechanisms of β-catenin are primarily transcriptional regulation, phosphorylation, and proteasomal degradation. Seven in absentia homolog (SIAH) is a member of the RING-finger-containing E3 ubiquitin ligases. SIAH is highly homologous to the *Drosophila melanogaster* seven in absentia (SINA) protein^18^. In *Homo sapiens*, two highly conserved homologs (Siah-1 and Siah-2) have been found^19^. In mammalian cells, SIAH1 can ubiquitinate many important target proteins, including the cell surface receptor DCC^20^, transcriptional regulators c-MYB^21^, POU2AF1^22^, and PML^23^, and the anti-apoptotic protein BAG1^24^. Importantly, SIAH1 causes indirect degradation of β-catenin through the formation of a complex with Siah-interacting proteins, Skp1 and Ebi^22,23^. SIAH1 expression is negatively correlated with tumor malignancy in human cancers and can induce cell cycle arrest and suppress tumor formation^25,26^. However, the regulatory mechanisms of SIAH1 expression in GBM remain unknown.

In this study, we demonstrated that PHF19 regulates GBM cell migration and invasion through a β-catenin-mediated signaling pathway. We found that PHF19 is involved in the regulation of β-catenin stability via inhibition of SIAH1 transcription. Our studies showed that PHF19 may be a potential therapeutic target for GBM treatment.

## Results

### High PHF19 expression is associated with poor prognosis in GBM patients

**Fig. 1:**
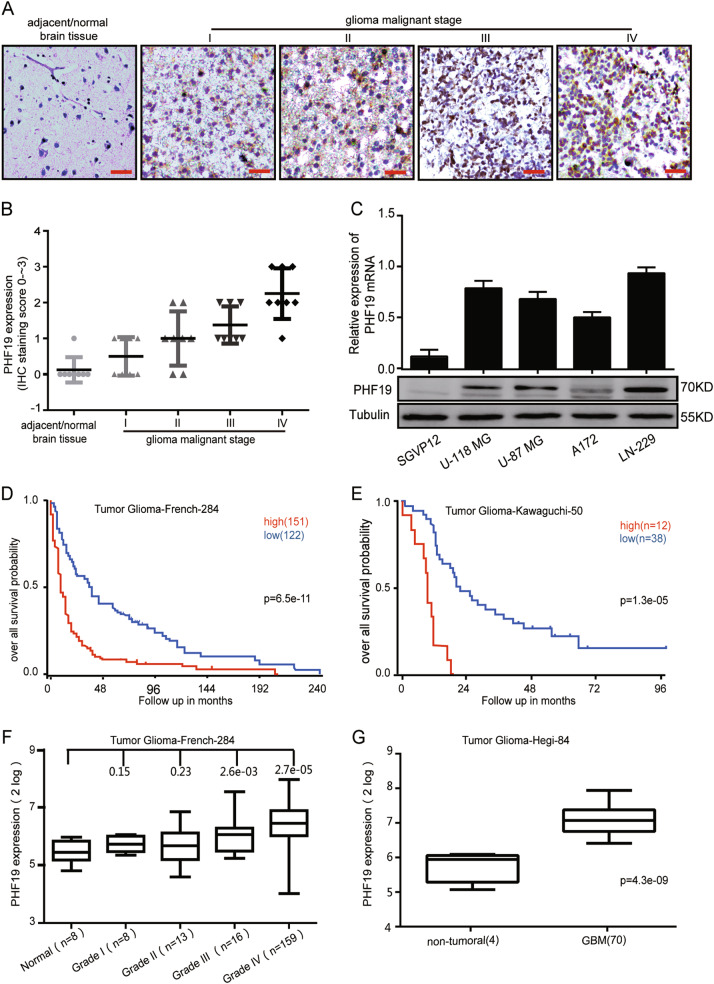
High PHF19 expression is associated with poor prognosis in patients with glioblastoma. **a** Representative immunohistochemical assays of PHF19 expression in human glioma tumors (I, II, III, IV) and adjacent normal brain tissue (lift). PHF19 expression in 70 glioma samples and 8 paired samples of normal tissue (I-8, II-8, III-8, IV-8) was assessed by immunohistochemistry. **b** Immunohistochemistry analyses of PHF19 expression levels in 70 glioma tumors and 10 normal tissue samples. **c** Western blot assay and q-PCR were performed to detect PHF19 expression in five cell lines including SGVP12, U-87 MG, U-118 MG, A172, and LN-229. **d**, **e** Results of the Kaplan–Meier analysis of progression-free survival and the log-rank test *P* values are indicated for the Tumor Glioma-French-284 dataset (left) and the Tumor Glioma-Kawaguchi-50 dataset (right). **f** Top left, box plot of PHF19 expression levels in peritumoral tissues (Normal) and grade I–IV gliomas. **g** Box plot of PHF19 expression levels in peritumoral tissues (Normal) and GBM in the Tumor Glioma Hegi 84 dataset with the log-rank test *P* values indicated

To determine whether the PHF19 is a prognostic factor for poor survival, we performed immunohistochemical staining on primary tissue microarray samples from glioma patients. The results showed that PHF19 expression levels were significantly increased in the advanced stages of glioma compared to those in adjacent normal tissues (Fig. 1a, b). Then, we examined PHF19 expression in four GBM cell lines (U-118 MG, U-87 MG, A172, and LN-229) and normal astrocytomas (SGVP12). We found that PHF19 was commonly expressed in the four GBM cell lines (Fig. 1c). To further confirm whether PHF19 is a prognostic marker for GBM, we analyzed the survival data from two databases (Tumor Glioma-French-284 and Tumor Glioma-Kawaguchi-50), which are available from the online R2 genomics analysis and visualization platform. Both the databases showed that high PHF19 expression is strongly associated with poor overall survival (Fig. 1d, e). Moreover, Kaplan–Meier analysis demonstrated that PHF19 expression levels were positively associated with glioma grade (Fig. 1f, g). Taken together, our data indicated that PHF19 is highly expressed in GBM and that high levels of PHF19 are associated with poor prognosis for GBM patients.

### PHF19 promotes cell proliferation and increases chemosensitivity of GBM

**Fig. 2:**
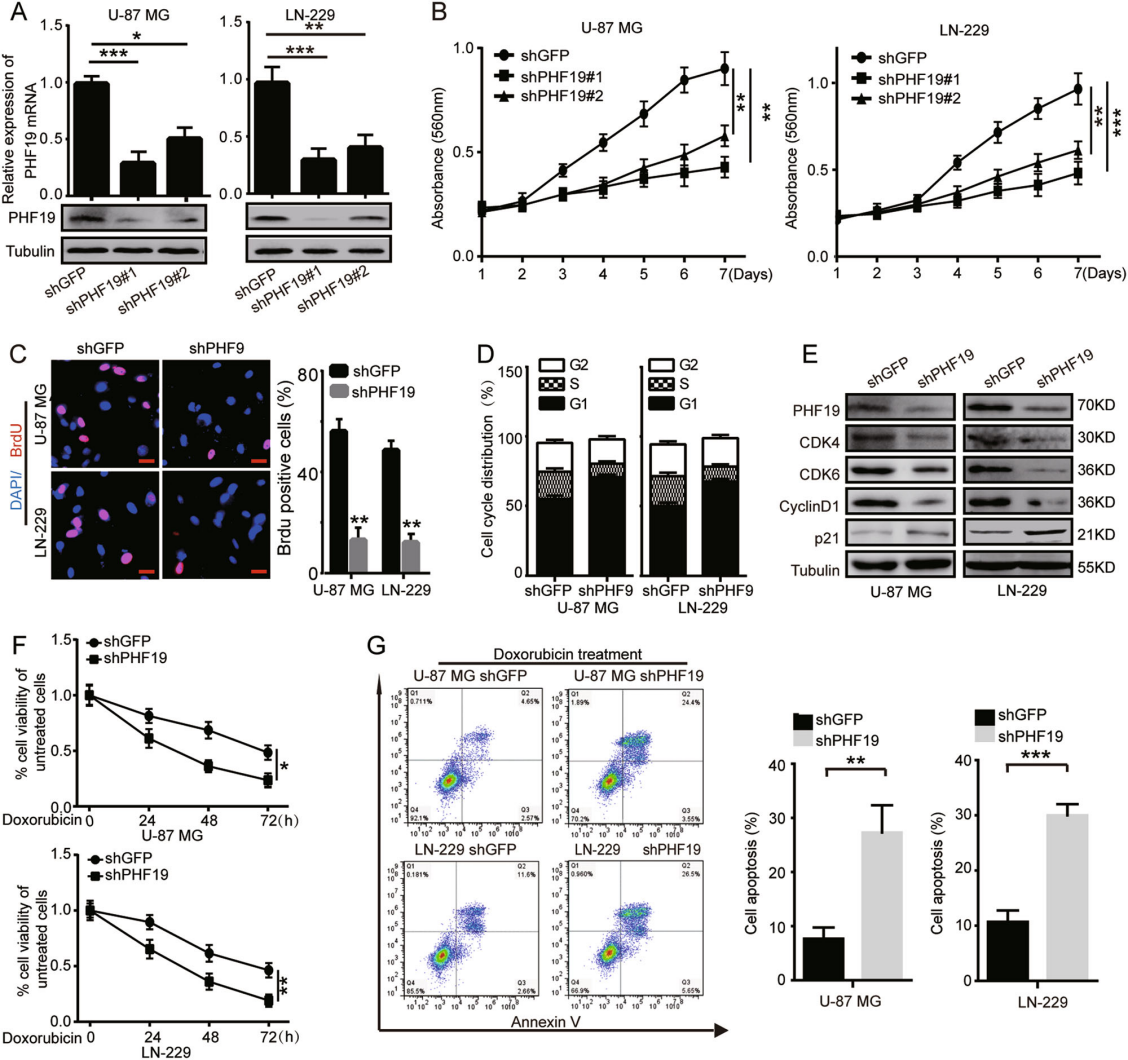
PHF19 promotes cell proliferation and increases chemosensitivity of GBM. **a** After PHF19 knockdown by shRNA in two glioblastoma cell lines, PHF19 expression was detected using qRT-PCR and Western blot analysis. **b** PHF19 knockdown inhibited the proliferation of U-87 MG and LN-229 cells. MTT assay was performed to examine the effect of PHF19 knockdown on cell viability. **c** BrdU assays were performed after PHF19 knockdown. Representative images show immunofluorescence and the quantification of BrdU-positive cells (scale bars, 20 mm). Data were analyzed using two-tailed Student *t* tests. Error bars, SEM, *n* = 3, ***P < 0.001. **d** We analyzed the cell cycle distribution of U-87 MG and LN-229 cells by flow cytometry. Error bars, SEM, *n* = 3, ***P<0.001,**P<0.01 . **e** Western blot assay was performed to detect the expression of G1 cell cycle regulatory proteins in PHF19-knockdown cells. **f** U-87 MG and LN-229 cells infected by shGFP or shPHF19 were exposed to 500 ng/mL doxorubicin for 0, 24, 48, and 72 h. **g** Apoptosis was assessed by flow cytometry

To investigate the role of PHF19 in GBM cell proliferation, we knocked down PHF19 by using two independent short hairpin RNA (shRNA) sequences against PHF19 in GBM cell lines (U-87 MG and LN-229), which were named shPHF19 #1 and shPHF19 #2. Western blot analysis showed that shPHF19 #1 exhibited the most significant reduction in PHF19 (Fig. 2a). 3-[4,5-dimethylthiazol-2-yl]-2,5 diphenyl tetrazolium bromide (MTT) assays also demonstrated that shPHF19 #1 resulted in a significant decrease in growth curve (Fig. 2b). Hence, the following experiments were all performed using the highly effective shPHF19 #1, which was used as a representative shPHF19, and short hairpin green fluorescence protein (shGFP) was used as a negative control. Bromodeoxyuridine (BrdU) assays were performed to show that PHF19 knockdown led to a significant reduction in DNA synthesis compared with that of the control cells (Fig. 2c). Then, we examined the cell cycle distribution of PHF19 knockdown cells and control cells by flow cytometry and found that PHF19 knockdown induced cell cycle arrest at the G1/S phase (Fig. 2d). To explore the molecular mechanisms underlying PHF19-induced cell cycle arrest, we detected several G1/S phase-related proteins. We found that the expression levels of CDK4, CDK6, and cyclin D were reduced, but that of p21 was increased following PHF19 knockdown (Fig. 2e). In addition, we investigated the function of PHF19 in GBM chemoresistance. We treated U-87 MG and LN-229 cells infected by shGFP and shPHF19 with doxorubicin. The results showed that PHF19 knockdown clearly increased doxorubicin-induced apoptosis in both U-87 MG and LN-229 cells (Fig. 2f, g).

To further confirm the effect of PHF19 on cell proliferation, we overexpressed PHF19 in PHF19-knockdown cells and found that PHF19 expression in U-87 MG and LN-229 cells was rescued (Supplementary Figure 1A). PHF19 overexpression rescued proliferation of the PHF19-knockdown cells, suggesting that the reduced cell proliferation caused by PHF19 knockdown is due to the decreased PHF19 expression in GBM cells (Supplementary Figure 1B, 1C, 1D, and 1E). In general, these results indicated that PHF19 is necessary for GBM cell growth and proliferation.

### PHF19 is essential for GBM cell migration and invasion

**Fig. 3:**
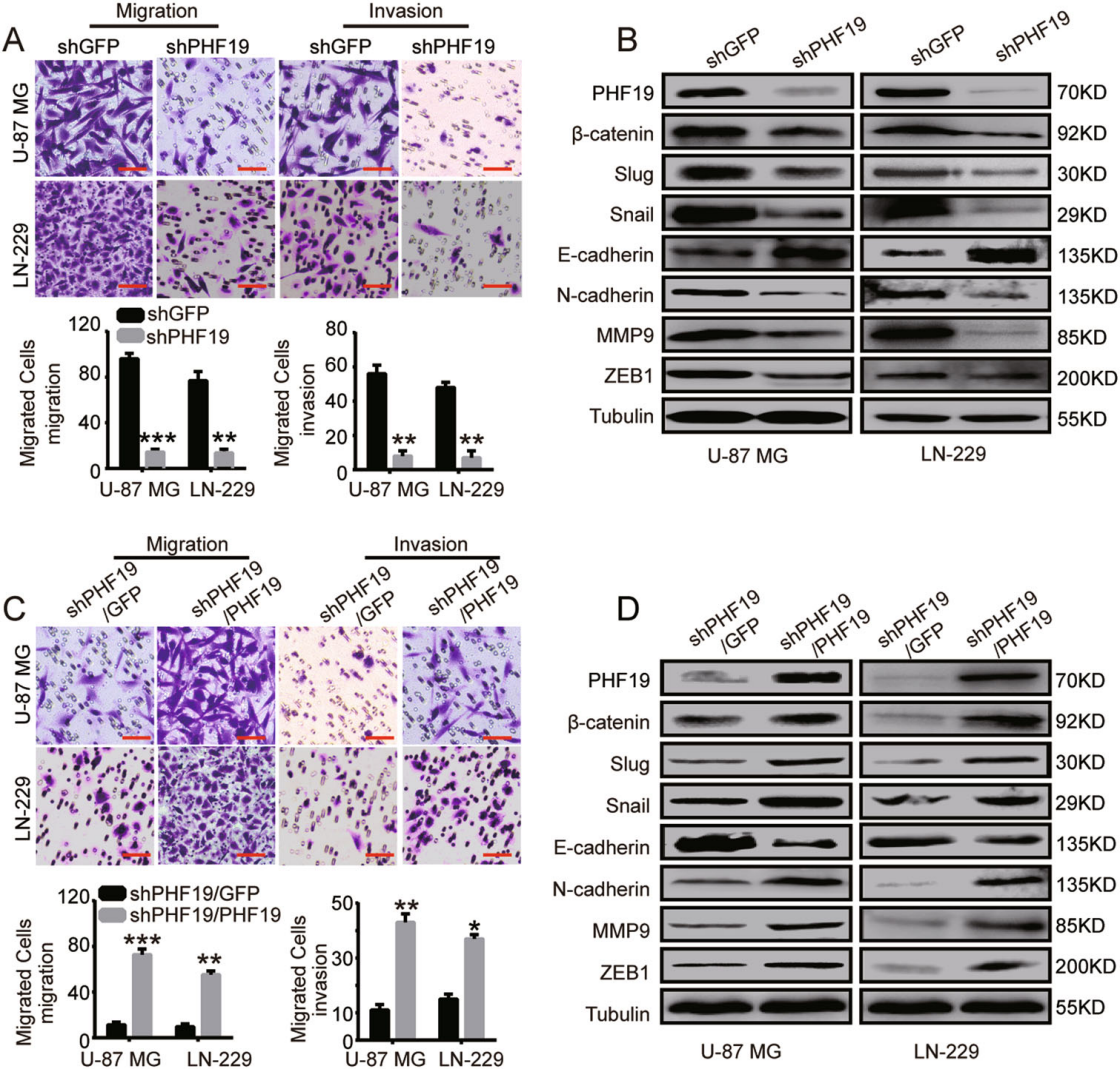
PHF19 is essential for GBM cell migration and invasion. **a** Migration and invasion assays were carried out in the PHF19-knockdown cells. **b** Western blot analysis was conducted to detect the expression level of key metastasis-related proteins in PHF19-knockdown cells. **c** Migration and invasion assays were carried out in PHF19-rescued PHF19-knockdown cells. **d** Western blot analysis was carried out to detect the expression level of key metastasis-related proteins in PHF19-rescued PHF19-knockdown cells. For Western blot analyses, tubulin served as the loading control. **P < 0.01, ***P < 0.001

As one of the most aggressive type of brain tumor cells, GBM cells have strong migratory and invasive abilities. To determine whether PHF19 is related to cell migration and invasion of GBM, we performed migration and invasion assays. Stable knockdown of PHF19 resulted in a significant reduction in the migration and invasion of GBM cells (Fig. 3a). To further explore the role of PHF19 in cell migration and invasion, we performed Western blot analysis to detect mesenchymal markers (Snail, Slug, ZEB1, and N-cadherin), an epithelial-to-mesenchymal transition biomarker (β-catenin), and an epithelial marker (E-cadherin). The cells with stable PHF19 knockdown showed decreased levels of Snail, Slug, ZEB1, N-cadherin, MMP9, and β-catenin, but E-cadherin was increased (Fig. 3b).

Restoration of PHF19 expression in PHF19-knockdown cells rescued their migratory and invasive abilities. Stable PHF19 overexpression in PHF19-knockdown cells resulted in the opposite trend (Fig. 3c). These observations suggested that PHF19 plays an important role in the regulation of metastasis and invasion in GBM cells (Fig. 3d).

### PHF19 is required for tumorigenesis and self-renewal of GBM cells

**Fig. 4:**
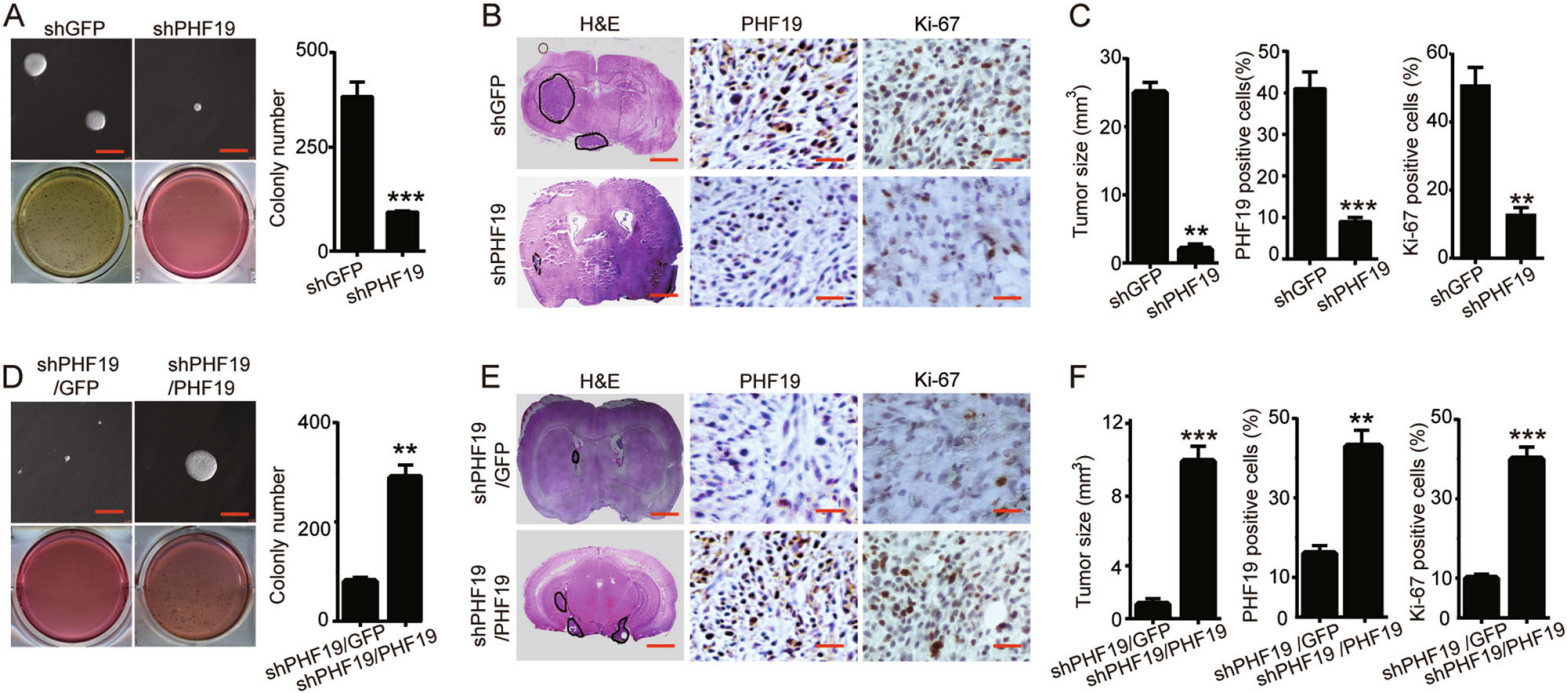
PHF19 is required for tumorigenesis and self-renewal of glioblastoma cells. **a** Soft agar assays were performed after PHF19 knockdown in the LN-229 cell line. The colony numbers were also quantified. **b** Orthotopic implantation was performed after PHF19 knockdown in LN-229 cells. Representative images of hematoxylin and eosin (H&E) staining (left) and immunohistochemical analysis of PHF19 expression (middle) and Ki-67 expression (right) are presented. **c** The quantification of the tumor size (left) immunohistochemistry analysis of Ki-67 expression (middle) and PHF19 expression (right) is presented. **d** The effects of PHF19 on the colony formation ability of the PHF19-rescued PHF19-knockdown LN-229 cells. **e** Orthotopic implantation was performed after PHF19 was restored in PHF19-knockdown LN-229 cells. Representative images of H&E staining (left) and immunohistochemical analysis of Ki-67 expression (middle) and PHF19 expression (right) are presented. **f** Effects of PHF19 restoration on tumor size (left) and immunohistochemical analysis of Ki-67 expression (middle) and PHF19 expression (right) after PHF19 restoration in PHF19-knockdown LN-229 cells. All data are shown as the means ± s.d, **P < 0.01 and ***P < 0.001. All *P* values are based on control versus treatment

To explore the roles of PHF19 in colony formation and tumor formation of GBM cells, we employed soft agar assays and orthotopic implantation, and the results showed that the colonies were smaller and fewer in the PHF19-knockdown U-87 MG and LN-229 cells than in the control cells (Fig. 4a). Next, orthotopic implantation experiments were performed to examine the effect of PHF19 on tumor growth in nonobese diabetic/severe combined immunodeficiency (NOD/SCID) mice. As shown in Fig. 4b, tumor formation was significantly slower and tumors were smaller in NOD/SCID mice injected with PHF19-knockdown LN-229 cells compared with control mice. Immunohistochemical staining showed that PHF19 expression was substantially reduced in the PHF19-knockdown tumor samples. In addition, the expression levels of Ki-67, a well-known cell proliferation marker, were also significantly reduced in the shPHF19 tumor samples (Fig. 4b, c).

When PHF19 expression was restored in PHF19-knockdown LN-229 cells, the tumor formation and colony formation capacities were also restored (Fig. 4d). Correspondingly, PHF19 and Ki-67 levels were rescued in the tumor samples (Fig. 4e, f). These results suggested that PHF19 plays an important role in the tumorigenesis of GBM cells.

### PHF19 is closely related to ubiquitination degradation of β-catenin

**Fig. 5:**
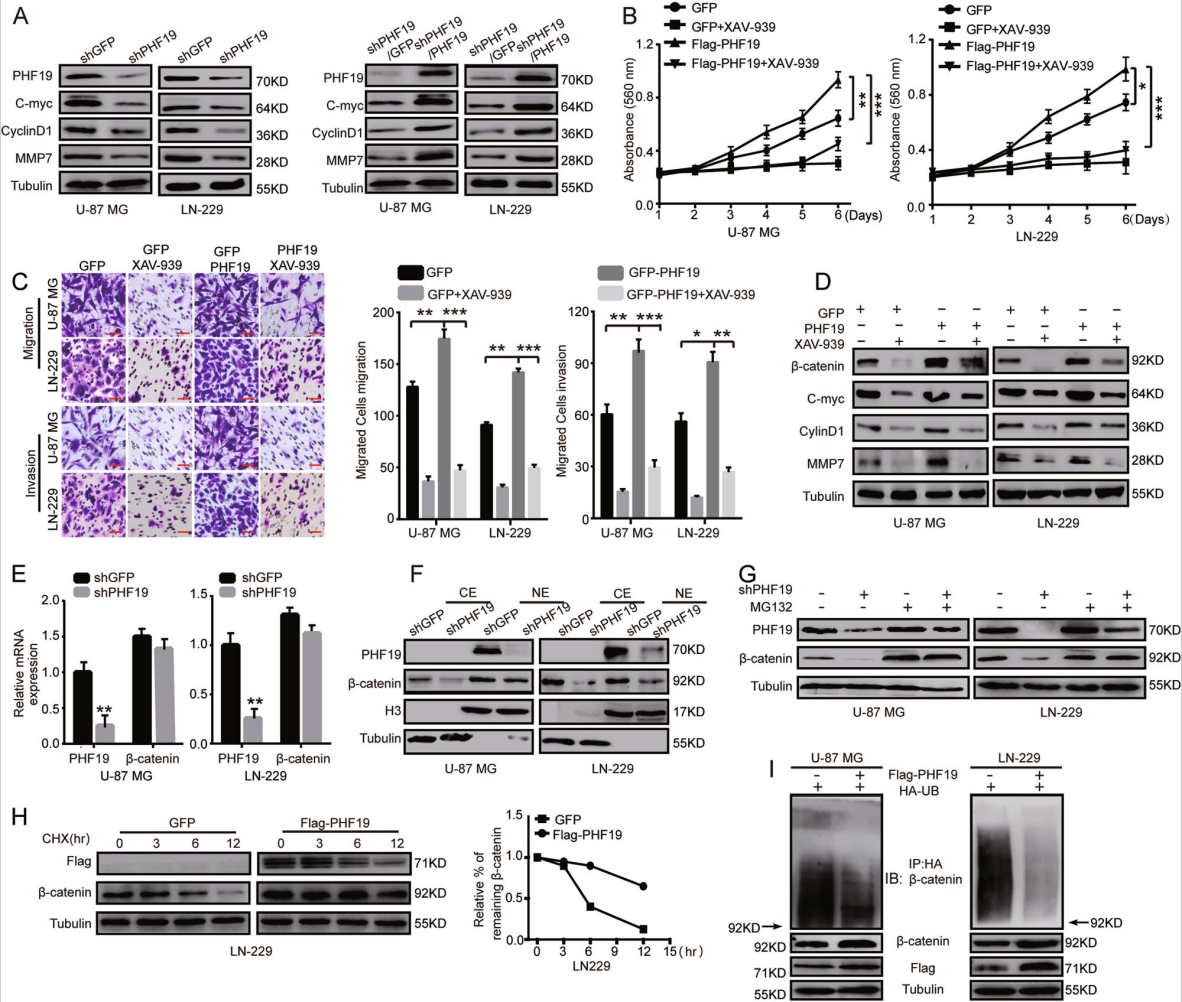
PHF19 is closely related to ubiquitination-mediated degradation of β-catenin. **a** Protein expression levels of PHF19, C-myc, cyclin D1, and MMP7 were analyzed by western blot analysis in PHF19-knockdown cells and restored cell lines. **b** MTT assays were performed to characterize the inhibitory effect of XAV-939 on the proliferation of PHF19-overexpressing cells. **c** Migration assays and invasion assays were performed to characterize the inhibitory effect of XAV-939 on the migration and invasion of PHF19-overexpressing cells. **d** Protein expression levels of β-catenin C-myc, cyclin D1, and MMP7 were analyzed by western blot analysis in U-87 MG and LN-229 cells treated with the β-catenin inhibitor XAV-939. **e** PHF19 and β-catenin expression was detected at the mRNA level using qRT-PCR. **f** Nuclear and cytoplasmic extraction assays were performed to detect the cytoplasmic and nuclear levels of PHF19 and β-catenin in PHF19-knockdown cells. **g** Cell lysates were prepared from the PHF19-knockdown cells that had been treated with or without MG-132 for 8 h. Equal amounts of cell lysates were immunoblotted with the indicated antibodies. **h** β-Catenin turnover rate of PHF19-overexpressing cells. LN-229 cells were transfected with Flag-PHF19 and were then treated with CHX (100 μg/mL) for the indicated times. Cell lysates were immunoblotted with the indicated antibodies. The turnover of β-catenin is indicated graphically. **i** The ubiquitinated β-catenin proteins were pulled down with an anti-HA antibody and immunoblotted with an anti-β-catenin antibody. All data are shown as the means ± s.d, **P < 0.01 and ***P < 0.001. All *P* values are based on control versus treatment

The previous data showed that β-catenin was downregulated in PHF19-knockdown cells (Fig. 3b). Thus, the downstream proteins of β-catenin were subsequently detected. Not surprisingly, C-myc, cyclin D1, and MMP7 levels were decreased as well, while they were restored after PHF19 expression was recovered (Fig. 5a). We hypothesized that PHF19 plays a regulatory role through β-catenin. To test this hypothesis, we used XAV-939, a Wnt/β-catenin signaling inhibitor, to treat PHF19-overexpressing cells. The promotion of cell proliferation, migration, and invasion was successfully blocked after XAV-939 treatment (Fig. 5b, c), suggesting that the proliferative and metastatic effects of PHF19 on GBM cells were β-catenin-dependent. The proteins downstream of β-catenin were analyzed by Western blot analysis in U-87 MG and LN-229 cells treated with the β-catenin inhibitor XAV-939 (Fig. 5d).

After we verified that β-catenin was downregulated after PHF19 knockdown, we further explored the mechanism by which β-catenin was decreased. Therefore, the messenger RNA (mRNA) level of β-catenin was first analyzed by reverse transcription-polymerase chain-reaction (RT-PCR). Relative to the western blot results, these analyses showed no significant changes in the mRNA level (Fig. 5e), suggesting that PHF19 may regulate β-catenin at the post-transcriptional level. To assess the relationship between PHF19 expression and β-catenin activation, we performed nuclear and cytoplasmic extraction assays. β-Catenin was substantially decreased in the cytoplasm but not obviously in the nucleus (Fig. 5f). Given that β-catenin was primarily degraded through the ubiquitin-proteasome pathway in the cytoplasm, we investigated whether the reduction of β-catenin protein level was due to PHF19-associated proteasomal degradation. Thus, the proteasome inhibitor MG-132 was used in our next assays. The decrease in β-catenin caused by PHF19 knockdown was abolished after MG-132 treatment (Fig. 5g). To confirm our results, we examined the turnover rate of PHF19 protein in the presence of cycloheximide (CHX), an inhibitor of protein synthesis. The turnover rate of PHF19 was enhanced in PHF19-expressing LN-229 cells compared with control plasmid-expressing cells (Fig. 5h) Taken together, these results demonstrated that PHF19 stabilized β-catenin by suppressing proteasomal degradation. Because ubiquitination is a pivotal step in the proteasomal degradation pathway, we tested whether PHF19 can regulate β-catenin ubiquitination. In vitro ubiquitination assays were carried out using Flag-PHF19. The results showed that the ubiquitination of β-catenin was significantly decreased after PHF19 overexpression (Fig. 5i). Taken together, these findings demonstrated that PHF19 increased β-catenin stability by reducing the ubiquitination of β-catenin.

### PHF19 regulates the transcription of SIAH1, a ubiquitination ligase of β-catenin

**Fig. 6:**
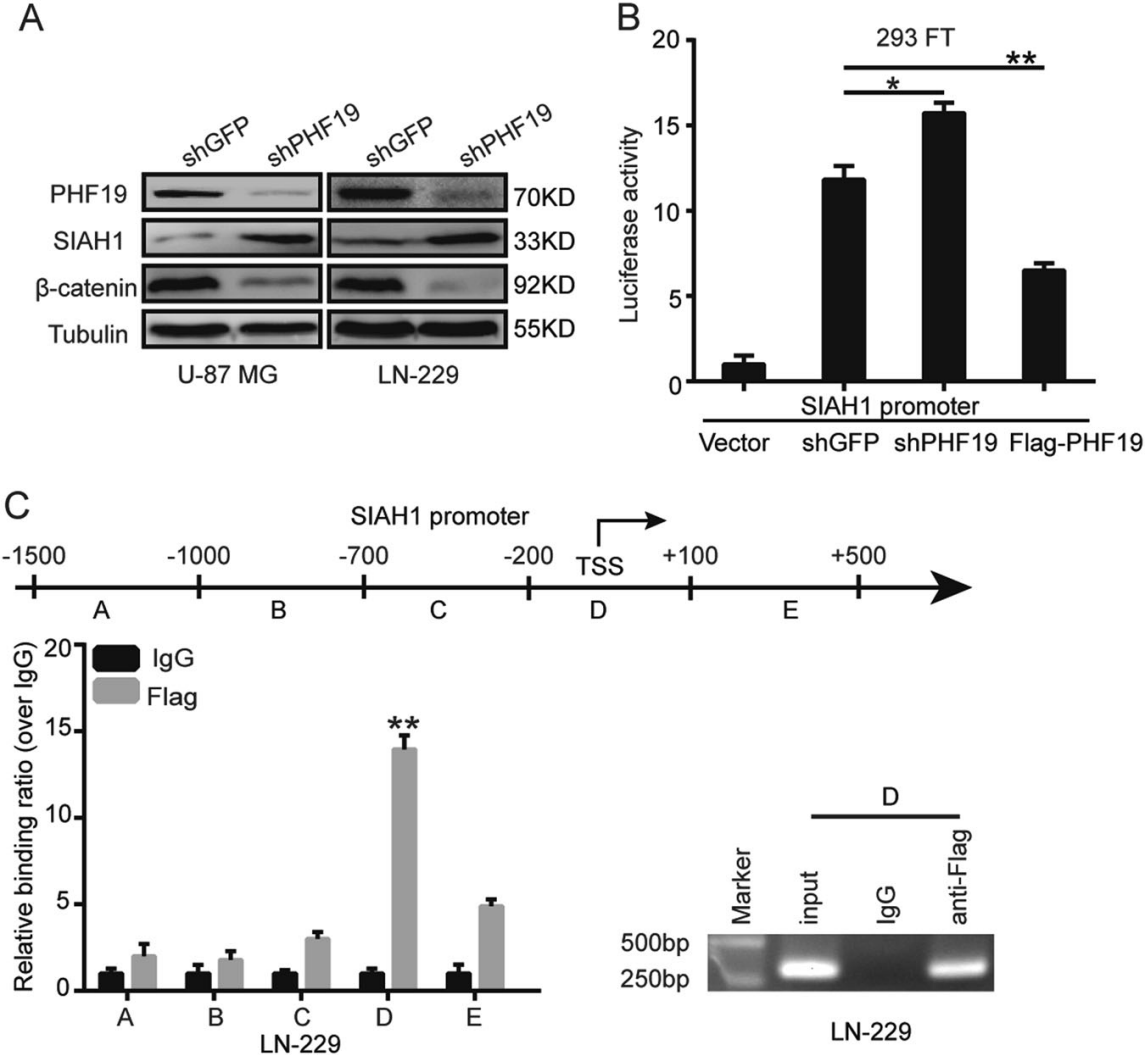
PHF19 regulates the transcription of SIAH1, which is the ubiquitin ligase for β-catenin. **a** We detected the expression of SIAH1 after knockdown of PHF19 by Western blotting. **b** The SIAH1 promoter regions were ligated into the pGL3 plasmid and co-transfected with pRL-TK/shPHF19/Flag-PHF19. Luciferase activity was examined 48 h after transfection. The pGL3-basic vector was used as the negative control. **c** A total of six sets of primers were designed within the human SIAH1 promoter, and a ChIP assay was performed using Flag antibodies. IgG was used as the negative control (left). Error bars, SEM; downregulation of SIAH1 in PHF19-knockdown cells abrogates the effects of PHF19 silencing. All data are shown as the means ± s.d, *P<0.05, **P < 0.01 and ***P < 0.001. All P values are based on control versus treatment

PHF19 is unable to mediate the ubiquitination of β-catenin directly because it is a transcriptional regulator. Thus, we examined the mRNA levels of β-catenin regulators, such as BTRC, SIAH1, and GSK3. Only SIAH1 was significantly upregulated when PHF19 was knocked down (Additional file 1: Figure S1A). As western blot analysis demonstrated that PHF19 knockdown decreased β-catenin expression by increasing SIAH1 expression (Fig. 6a), we hypothesized that SIAH1 might be a downstream effector of PHF19 in GBM cells. Next, a dual-luciferase reporter assay was designed to study the mechanism by which PHF19 regulates SIAH1 expression in PHF19-knockdown LN-229 cells. Fragments of SIAH1 promoter regions were designed and inserted into the pGL3-basic vector. We co-transfected the shPHF9/Flag-PHF19 plasmid and the SIAH1 promoter into 293FT cells. The empty pGL3-basic vector was used as the control. The results demonstrated that SIAH1 promoter activity was significantly increased by knocking down PHF19. Meanwhile, SIAH1 promoter activity was reduced when it was co-transfected with Flag-PHF19 (Fig. 6b). To determine whether the suppression of SIAH1 promoter activity was caused by PHF19 binding, we performed a chromatin immunoprecipitation (ChIP) assay to map the PHF19-binding locus on the SIAH1 promoter. As shown in Fig. 6c, PHF19-binding sites were enriched in proximal region D (−200 to +100 bp) of the promoter. This finding confirmed our previous results, showing that PHF19 inhibits SIAH1 transcription by directly binding to its promoter.

### Downregulation of SIAH1 in PHF19-knockdown cells abrogates the effects induced by PHF19 silencing

**Fig. 7:**
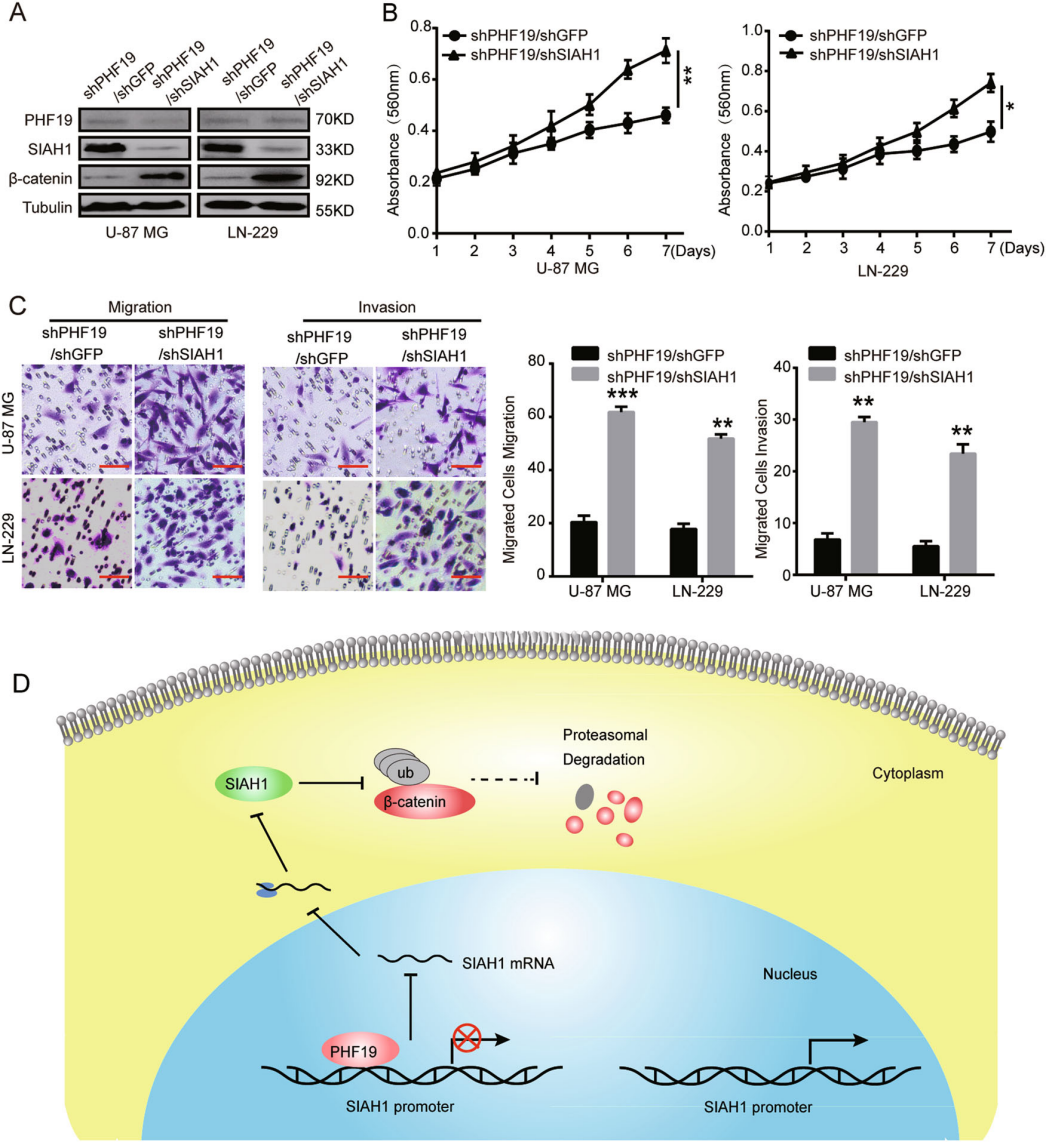
Downregulation of SIAH1 in PHF19-knockdown cells abrogates the effects of PHF19 silencing. **a** Western blot assay was performed to detect SIAH1 and β-catenin protein levels after SIAH1 knockdown by shRNA following PHF19 downregulation. **b** The effects of SIAH1 downregulation on the proliferation of PHF19-knockdown cells. **c** The effects of SIAH1 downregulation on the migration and invasion of PHF19-knockdown cells. **d** Model of the impact of the PHF19-SIAH1 axis on the regulation of β-catenin signaling. All data are shown as the means ± s.d., *P < 0.05, **P < 0.01 and ***P <0.001 . All *P* values are based on control versus treatment

As Western blot analysis demonstrated that PHF19 knockdown decreased β-catenin expression by increasing SIAH1 expression (Fig. 6a), we hypothesized that SIAH1 might be a downstream effector of PHF19 in GBM cells. To confirm our hypothesis, we knocked down SIAH1 expression with the highly effective shSIAH1 #1 in PHF19-knockdown cells, inducing SIAH1 downregulation. Western blot analysis showed that β-catenin expression was elevated after SIAH1 knockdown in PHF19-knockdown cells (Fig. 7a). Simultaneously, the proliferation, migration, and invasion of PHF19-knockdown cells were significantly increased after SIAH1 downregulation (Fig. 7b, c). In conclusion, these results demonstrated that SIAH1 was a pivotal downstream effector of PHF19 and that the PHF19-SIAH1 axis regulated cell growth, migration, invasion, and tumorigenicity via a β-catenin-mediated signaling pathway in GBM (Fig. 7d).

In conclusion, these results demonstrated that SIAH1 is a pivotal downstream effector of PHF19 and that the PHF19–SIAH1 axis regulates cell growth, migration, invasion, and tumorigenicity via a β-catenin-mediated signaling pathway in GBM.

## Discussion

GBM is the most deadly form of cancer of the human central nervous system, with a median survival of 12–15 months and a 5-year survival rate of <5%^27^. An increasing number of studies in recent decades have attempted to illuminate the mechanisms underlying the unlimited growth and metastasis of GBM cells. PHF19, as a PRC2-associated factor that forms sub-complexes with PRC2 core components, is upregulated in some human cancers^12,28^. Previous studies have shown that PHF19 plays important roles in cancer progression, including cell cycle regulation, proliferation, invasion, and drug resistance^14,29^. Recently, PHF19 has been found to be highly expressed in GBM^30^, but the biological function of PHF19 in GBM is not clear, and the molecular mechanisms that underlie its effects remain uncertain. In this study, we demonstrated that high expression of PHF19 indicates poor prognosis of GBM patients.

It has been reported that PHF19 regulates gene expression by affecting the stability of the PRC2 complex. Western blot analyses were performed to detect the expression of SUZ12, EZH2, and the core components of PRC2. These analyses showed no significant changes in the levels of these proteins. PHF1 is also known to affect cell cycle progression by regulating p53 stability, which is independent of PRC2. Therefore, we believe that PHF19 independently influences the progression of GBM cells and does not rely on PRC2.

Using shRNA-mediated gene silencing techniques, we successfully knocked down PHF19 in GBM cells. We demonstrated that PHF19 knockdown inhibited GBM cell proliferation and metastasis. We added doxorubicin to shPHF19 cells and found that the cells were more sensitive to this agent, which induces apoptosis through DNA damage. Recent studies have shown that PHF19 affects DNA damage in HeLa and pluripotent stem cells. Therefore, we speculate that the role of PHF19 in GBM cells may be related to DNA damage.

PHF19 silencing attenuated the expression of several genes involved in cell proliferation (cyclin D1, C-myc) and metastasis (MMP7), all of which are target genes of β-catenin. β-Catenin is a pluripotency protein that plays a crucial role in developmental processes, self-renewal, and tumorigenesis via its effects on Wnt-dependent signal transduction and E-cadherin-mediated cell adhesion^31^. When we knocked down PHF19, we found that the protein levels of β-catenin and its downstream genes changed significantly. Further investigation revealed that the level of cytoplasmic β-catenin protein was significantly reduced, but its total mRNA level was not changed. Cytoplasmic β-catenin generally undergoes ubiquitination and degradation, and we therefore determined whether PHF19 regulates ubiquitin-mediated degradation of β-catenin. Unexpectedly, PHF19 was found to regulate the ubiquitination of β-catenin. We next examined the expression of ubiquitin ligases correlated with β-catenin, such as BTRC and SIAH1^32^. Among these E3 ubiquitin ligases, only SIAH1 was significantly upregulated after knockdown of PHF19. Subsequently, dual-luciferase reporter assays and ChIP assays suggested that PHF19 regulated the transcription of SIAH1 through binding to its promoter.

Downregulation of PHF19 expression affected cell proliferation and migration. Whether this effect is mediated through the SIAH1–β-catenin axis alone remains to be determined.

In summary, we identified PHF19 as an oncogene with prognostic significance in GBM. PHF19 promotes GBM progression via a non-canonical mechanism, which blocks the degradation of β-catenin through transcriptional suppression of SIAH1. Our studies provide insights into the applicability of using the PHF19–SIAH1–β-catenin axis as a potential therapeutic target in cancer. In subsequent studies, we aim to identify the definite binding sequence in the SIAH1 promoter.

## Materials and methods

### Cell lines, reagents, and antibodies

All GBM cell lines and normal SVGP12 cells were originally purchased from ATCC (American Type Culture Collection, Rockville, MD, USA). Cells were cultured in DMEM/DME/F-12 1:1 (Dulbecco's modified Eagle's medium/Ham's nutrient mixture F-12) (Invitrogen) supplemented with 10% fetal bovine serum (FBS) and 1% penicillin and streptomycin (P/S). All cell lines were incubated in a humidified atmosphere at 37 °C and 5% CO_2_. The growth media, reagents, and supplements were obtained from Gibco. HA (H3663) and Flag (SAB4200071) antibodies, MG-132 (M7449), and CHX (C7698) were acquired from Sigma (Shanghai, China); PHF19 (77271 S), β-catenin (ab32572), MMP7 (ab38996) antibodies and the Cell Cycle Antibody Sampler Kit (no. 9932), the Cyclin Antibody Sampler Kit (no. 9869), and the Epithelial–Mesenchymal Transition Antibody Sampler Kit (no. 9782) were purchased from Cell Signaling Technology (Shanghai, China). SIAH1 (ab2237), C-myc (ab32), and Axin2 (ab109307) antibodies were obtained from Abcam (Shanghai, China). All antibodies were diluted according to the instructions.

### Transfection and Infection

PHF19-specific shRNA, SIAH1-specific shRNA, and GFP-specific shRNA (shGFP) were purchased from Addgene and inserted into the pLKO.1 vector. PHF19 Stealth Select RNAi was used (Supplementary Table 1). shSIAH1 #1 (Supplementary Table 1), a plasmid encoding human PHF19, was constructed by PCR-based amplification and subsequently subcloned into the pCDH-CMV-MCS-EF1-copGFP vector. A plasmid encoding HA-tagged-ubiquitin were obtained from Addgene (Beijing, China). The plasmid encoding Flag-tagged PHF19 was purchased from Addgene (Beijing, China). The 293FT cell lines were co-transfected with the packaging plasmids pLP1, pLP2, and pLP/VSVG (Invitrogen) and the corresponding shRNA plasmids or overexpressing plasmids to produce lentivirus. Lipofectamine 2000 was used for all transfections according to the manufacturer’s instructions. At 48 h after transfection, the virus supernatant was collected to infect GBM cells, with a final concentration of 4 μg/mL polybrene. A resistant cell line was selected using 2 mg/mL puromycin for use in subsequent experiments.

### Immunohistochemistry staining

The immunological tissue used in the experiment was purchased from Alenabio. Paraffin-embedded tumor specimens were cut into 5 mm slices and then deparaffinized and rehydrated. The sections were immersed in 10 mM citrate buffer (pH = 6.0) and heated in a microwave oven for 20 min for antigen repair. After quenching endogenous peroxidase activity and blocking with normal goat serum, the slices were incubated with primary antibodies (PHF19, Ki-67) followed by horseradish peroxidase-linked secondary antibody. Slices were visualized with (diaminobenzidine) DBA and counterstained with hematoxylin before observing with a microscope.

### Cell proliferation analysis

The MTT assay was used to analyze cell growth curves. To determine cell viability, 1 × 10^3^ cells were placed in flat-bottomed 96-well plates for 6 days, and the MTT assay was performed at the indicated time points from days 0 to 6, according to the manufacturer’s instructions. All experiments were repeated three times independently.

### BrdU staining

For BrdU staining, 1 × 10^4^ cells were cultured on coverslips in 24-well plates. The cells were incubated with 10 μg/mL BrdU (Sigma) for 30 min, washed with phosphate-buffered saline (PBS), and fixed with 4% paraformaldehyde for 15 min. Then, samples were blocked with 5% goat serum for 2 h, followed by incubation with a primary antibody against BrdU (1:200, ab6326, Abcam, Cambridge, MA, USA) for 1 h, and then an Alexa Fluor^®^ 594 secondary antibody (H + L; Invitrogen). DAPI (4′,6-diamidino-2-phenylindole) (300 nM) was used for nuclear staining; the percentage of BrdU incorporation was calculated from at least 10 randomly selected fields.

### Flow cytometry

For cell cycle analysis, 1 × 10^6^ cells were harvested and fixed in ice-cold 70% ethanol overnight at 4 °C, incubated with propidium iodide (PI), and analyzed by flow cytometry (BD Biosciences, San Jose, CA, USA) with Cell Quest software (BD Biosciences).

### Sub-G1 analysis assay

Treated cells, including the medium, were collected and centrifuged at 600 × *g* for 5 min, and the supernatant was removed. Cells were washed twice with PBS and fixed with 70% ice-cold ethanol for 24 h. Then, the cells were centrifuged and washed again and adjusted to a final concentration of 1 × 10^6^ cells per mL. Next, 50 mg/mL PI and 0.25 ml RNase (1 mg/mL in PBS) were added to a 0.25 ml cell sample. The cells were incubated in the dark at 4 °C for 30 min before flow cytometric analysis (BD Accurri C6 cytometer and Modifit LT3.3). In the DNA histogram, the amplitude of the sub-G1 DNA peak represents the number of apoptotic cells. The experiment was repeated at least three times.

### Migration, invasion assay

For the migration and invasion assay, cells were seeded in the upper chambers (8 μm pore size; Corning, Beijing, China) of a 24-well transwell. The membranes were painted with Matrigel (BD Biosciences) in the invasion assay. Cells with serum-free media were added to the upper chamber. The lower chambers were filled with culture medium supplemented with 10% FBS. At 48 h after incubation, the cells were fixed in 4% paraformaldehyde for 15 min and then incubated with crystal violet. The mean number of cells were calculated from more than 5 randomly chosen microscopic fields per filter and visualized under bright-field microscopy.

### Western blot analysis

Cells were lysed in lysis buffer containing 50 mM Tris-HCl, pH 7.5, 150 mM NaCl, 1% Nonidet P-40, 0.25% sodium deoxycholate, 0.1% sodium dodecyl sulfate (SDS) with a complete protease inhibitor cocktail (Roche) and phosphatase inhibitors (Sigma-Aldrich). Cell lysates were separated by SDS–polyacrylamide gel electrophoresis (SDS-PAGE) and were transferred to a polyvinylidene difluoride membrane. SDS-PAGE gels were calibrated using Magic Mark XP Western Standard (Invitrogen). After blocking with skim milk for 2 h, the membrane sections were incubated with the primary antibody and the corresponding secondary antibody. Primary antibodies were used at a dilution of 1:1000. Secondary antibodies (peroxidase-labeled anti-mouse and anti-rabbit antibodies) were used at a dilution of 1:5000. Finally, bound antibodies were captured by chemiluminescence using the ECL Prime Western blotting (WB) detection system (GE Healthcare), and luminescent images were analyzed with a Lumino Imager (LAS- 4000 mini; Fuji Film Inc.).

### Ubiquitination assay

For the in vivo ubiquitination assay, 293FT cells were co-transfected with the indicated plasmids. At 48 h after transfection, cells were treated with 50 μg/mL of the proteasome inhibitor MG-132 for 6 h. Cells were then lysed in Cell Lysis Buffer (Sigma) for western blotting and immunoprecipitation (IP) following the same protocol used in co-IP.

### Turnover assay

Cells were transfected with the indicated plasmids. CHX was added to media at a 50 μg/mL final concentration. The cells were harvested at the indicated time points after CHX treatment, and protein levels were analyzed by immunoblotting. The protein density was measured using a densitometer, and the integrated optical density was calculated.

### Soft agar assay

Low-melting-point agar was used as the solidified bottom layer in a 6-well plate. A total of 1 × 10^3^ cells were mixed with 0.3% agar in growth medium. After 14–21 days of culture, the colonies in each well were photographed and recorded.

### Xenograft assay

Five-week-old male nude mice were housed in SPF rooms and maintained under constant temperature and humidity. Human GBM cell lines (LN-229) cells (1 × 10^5^ cells) stably transfected with shGFP, shPHF19, shPHF19/GFP, or shPHF19/PHF19 were slowly injected into the brain of each mouse. At the termination of the experiment, the brains were collected and soaked in 4% polyoxymethylene. Single blinding and randomization were used for the measurements. All experiments were approved by the Animal Care and Use Committee of Southwest University and were performed according to the “Guidelines for Animal Care and Use” (Ministry of Science and Technology of China, 2006).

### Quantitative and RT-PCR

Total RNA was extracted from cells using TRIzol reagent (Invitrogen) according to the manufacturer’s protocol. Then, 2 μg of RNA was reverse transcribed into complementary DNA for each sample. mRNA expression was based on Ct values and was normalized to the values of glyceraldehyde-3-phosphate dehydrogenase as a control. The relevant primer sequences are presented in Supplementary Table 2.

### Luciferase reporter assay

The SIAH1 promoter fragment was amplified using PCR and ligated into the PGL3-basic vector (Promega). The empty PGL3-basic vector served as the negative control. A total of 1.5–3 × 10^5^ cells per well were placed in 24-well plates for cell transfection. A total of 1 mg pGL3 plasmid, 100 ng pRL-TK internal control vector (Promega), and shPHF19/Flag-PHF19 vector were co-transfected into 293FT cells in serum-free Opti-MEM Reduced Serum Medium (Life Technologies). After 4–6 h, the culture medium was added to each well to a final volume of 1 ml. After a further incubation of 48 h, a luciferase reporter assay was performed according to the manufacturer’s instructions (Promega). Luciferase activity was normalized to pRL-TK activity. Each experiment was performed in triplicate.

### Chromatin immunoprecipitation

A ChIP assay was performed using a ChIP Assay Kit (Millipore) according to the manufacturer’s instructions. Briefly, LN-229 cells were cross-linked and lysed, and DNA was sheared into 200–800 bp fragments using sonication. Precleared chromatin was immunoprecipitated with a Flag antibody (Santa Cruz), and DNA was isolated after reverse cross-linking for quantitative real-time PCR (qRT-PCR). The relevant primer sequences are presented in Table S3 in the Supplemental Materials. ChIP analyses were then performed as previously described^33^.

### Patient data analysis and patient tumor tissues

Patient data and gene expression data sets were obtained from the R2: microarray analysis and visualization platform (http://hgserver1.amc.nl/cgibin/r2/main.cgi). Kaplan–Meier analysis and survival curves were carried out using GraphPad Prism (version 6.0, GraphPad Software, San Diego, CA, USA). All cutoff values for separating high and low expression groups were determined by the online R2 database algorithm. Tissue analysis was approved by the Ethics Committee of the Southwest University of China. All the patients provided written informed consent to participate.

### Statistical analysis

All experiments were carried out in triplicate according to statistical parameters including sample size and significance analysis, as shown in the figure. A two-tailed Student's *t* test was performed to calculate the significance. Normally distributed data are expressed as the 95% confidence level range, whereas similar quantitative data are expressed as the mean ± s.d. *P* < 0.05 values were considered statistically significant.

## Electronic supplementary material

supplementary figure legends

supplement-1

supplement-2

table

## References

[1] Thakkar JP (2014). Epidemiologic and molecular prognostic review of glioblastoma. Cancer Epidemiol. Biomark. Prev..

[2] Osuka S, Van Meir EG (2017). Overcoming therapeutic resistance in glioblastoma: the way forward. J. Clin. Invest..

[3] Dunn GP (2012). Emerging insights into the molecular and cellular basis of glioblastoma. Genes Dev..

[4] Wen PY, Kesari S (2008). Malignant gliomas in adults. N. Engl. J. Med..

[5] Cruickshanks Nichola, Zhang Ying, Yuan Fang, Pahuski Mary, Gibert Myron, Abounader Roger (2017). Role and Therapeutic Targeting of the HGF/MET Pathway in Glioblastoma. Cancers.

[6] Lee I (2017). Advances in surgical approaches in glioblastoma (GBM). Chin. Clin. Oncol..

[7] Sauvageau M, Sauvageau G (2010). Polycomb group proteins: multi-faceted regulators of somatic stem cells and cancer. Cell Stem Cell.

[8] Wang S, Robertson GP, Zhu J (2004). A novel human homologue of *Drosophila* polycomblike gene is up-regulated in multiple cancers. Gene.

[9] Ballare C (2012). Phf19 links methylated Lys36 of histone H3 to regulation of Polycomb activity. Nat. Struct. Mol. Biol..

[10] Boulay G (2011). Functional characterization of human Polycomb-like 3 isoforms identifies them as components of distinct EZH2 protein complexes. Biochem. J..

[11] Brien GL (2012). Polycomb PHF19 binds H3K36me3 and recruits PRC2 and demethylase NO66 to embryonic stem cell genes during differentiation. Nat. Struct. Mol. Biol..

[12] Xu H (2015). MicroRNA-195-5p acts as an anti-oncogene by targeting PHF19 in hepatocellular carcinoma. Oncol. Rep..

[13] Murakami H (2013). Establishment of new intraperitoneal paclitaxel-resistant gastric cancer cell lines and comprehensive gene expression analysis. Anticancer Res..

[14] Callahan R (2012). Genes affected by mouse mammary tumor virus (MMTV) proviral insertions in mouse mammary tumors are deregulated or mutated in primary human mammary tumors. Oncotarget.

[15] Zheng H (2010). PLAGL2 regulates Wnt signaling to impede differentiation in neural stem cells and gliomas. Cancer Cell.

[16] Denysenko T (2016). WNT/beta-catenin signaling pathway and downstream modulators in low- and high-grade glioma. Cancer Genom. Proteom..

[17] Valenta T, Hausmann G, Basler K (2012). The many faces and functions of beta-catenin. EMBO J..

[18] Kramer OH (2013). SIAH proteins: critical roles in leukemogenesis. Leukemia.

[19] House CM, Moller A, Bowtell DD (2009). Siah proteins: novel drug targets in the Ras and hypoxia pathways. Cancer Res..

[20] Li Q (2015). Central role of SIAH inhibition in DCC-dependent cardioprotection provoked by netrin-1/NO. Proc. Natl. Acad. Sci. USA.

[21] Imig J (2011). microRNA profiling in Epstein–Barr virus-associated B-cell lymphoma. Nucleic Acids Res..

[22] Boehm J (2001). Regulation of BOB.1/OBF.1 stability by SIAH. EMBO J..

[23] Pietschmann K (2012). Differential regulation of PML-RARalpha stability by the ubiquitin ligases SIAH1/SIAH2 and TRIAD1. Int. J. Biochem. Cell Biol..

[24] Sourisseau T (2001). Alteration of the stability of Bag-1 protein in the control of olfactory neuronal apoptosis. J. Cell Sci..

[25] Leung CO (2014). miR-135a leads to cervical cancer cell transformation through regulation of beta-catenin via a SIAH1-dependent ubiquitin proteosomal pathway. Carcinogenesis.

[26] Xie W (2009). E2F1 represses beta-catenin/TCF activity by direct up-regulation of Siah1. J. Cell. Mol. Med..

[27] Cloughesy TF, Cavenee WK, Mischel PS (2014). Glioblastoma: from molecular pathology to targeted treatment. Annu. Rev. Pathol..

[28] Pan MR (2015). G9a orchestrates PCL3 and KDM7A to promote histone H3K27 methylation. Sci. Rep..

[29] Ghislin S (2012). PHF19 and Akt control the switch between proliferative and invasive states in melanoma. Cell Cycle.

[30] Li G (2013). Altered expression of polycomb group genes in glioblastoma multiforme. PLoS ONE.

[31] Huber O, Bierkamp C, Kemler R (1996). Cadherins and catenins in development. Curr. Opin. Cell Biol..

[32] Ji L (2017). The SIAH E3 ubiquitin ligases promote Wnt/beta-catenin signaling through mediating Wnt-induced Axin degradation. Genes Dev..

[33] Wang F (2017). Morusin inhibits cell proliferation and tumor growth by down-regulating c-Myc in human gastric cancer. Oncotarget.

