# Supplementary material for: PHF19 promotes the proliferation, migration, and chemosensitivity of glioblastoma to doxorubicin through modulation of the SIAH1/β–catenin axis
Source: Cell Death Dis. 2018 Oct 15;9(11):1049. doi: 10.1038/s41419-018-1082-z (PMC6189144; doi:10.1038/s41419-018-1082-z)
Supplement: Supplementary file 1 — supplementary figure legends [file 41419_2018_1082_MOESM1_ESM.docx]

**supplementary figure legends**

Supplementary Figure 1. PHF19 overexpression nearly rescued proliferative ability (A, B) PHF19 overexpression was nearly able to rescue the proliferative ability of PHF19-knockdown cells. MTT assay was performed to examine the effect of PHF19 overexpression on the proliferation of PHF19-knockdown cells. (C) BrdU assays were performed after overexpression of PHF19 in PHF19-knockdown cells, and the summary graph of BrdU-positive cell number is shown. Error bars, SEM, n = 3, ***P＜001, **P＜0.05. (D) Flow cytometry assays were performed to examine cell cycle distribution in shPHF19/PHF19 cell lines. (E) Several G1 cell cycle regulatory proteins were analyzed by western blot.

### Supplementary Figure 2 (A) Several genes related to β-catenin degradation were detected by RT-PCR.
