# Supplementary figures and images for: PHF19 promotes the proliferation, migration, and chemosensitivity of glioblastoma to doxorubicin through modulation of the SIAH1/β–catenin axis
Source: Cell Death Dis. 2018 Oct 15;9(11):1049. doi: 10.1038/s41419-018-1082-z (PMC6189144; doi:10.1038/s41419-018-1082-z)

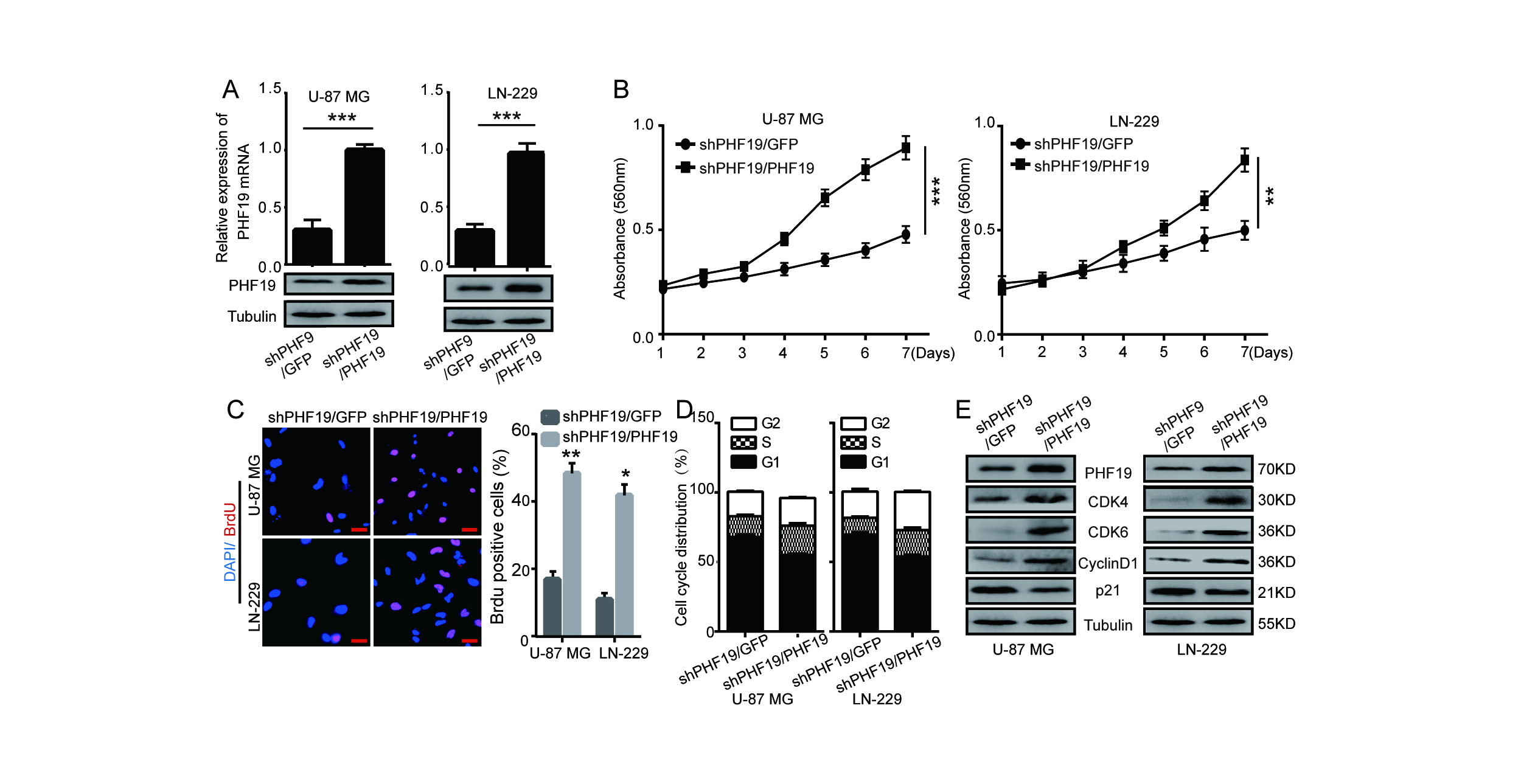

Supplement: Supplementary file 2 — supplement-1 [file 41419_2018_1082_MOESM2_ESM.tif]

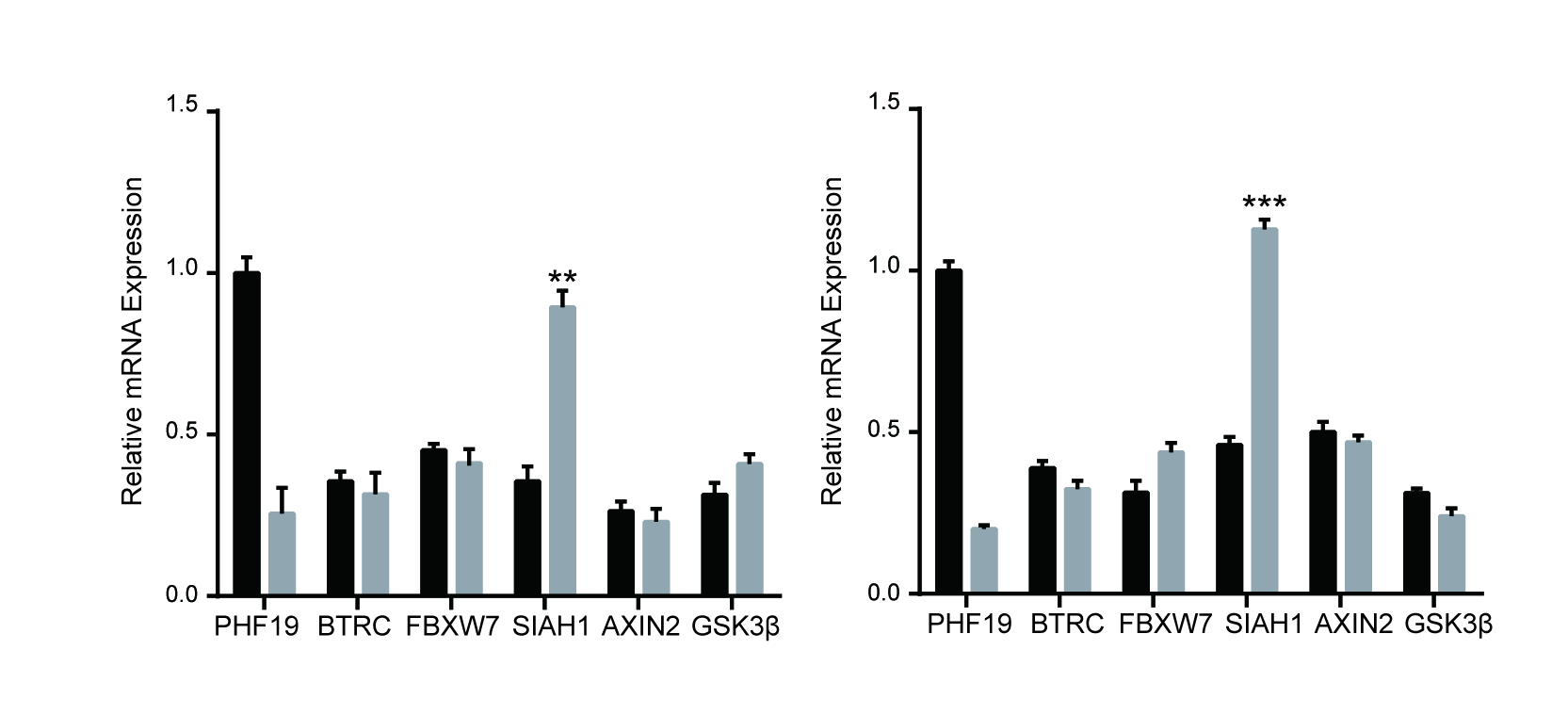

Supplement: Supplementary file 3 — supplement-2 [file 41419_2018_1082_MOESM3_ESM.tif]
