# Supplementary material for: PHF19 promotes the proliferation, migration, and chemosensitivity of glioblastoma to doxorubicin through modulation of the SIAH1/β–catenin axis
Source: Cell Death Dis. 2018 Oct 15;9(11):1049. doi: 10.1038/s41419-018-1082-z (PMC6189144; doi:10.1038/s41419-018-1082-z)
Supplement: Supplementary file 4 — table [file 41419_2018_1082_MOESM4_ESM.docx]

| shPHF19 #1-F | CCGGCTCGTGACTTTCGAAGATAATCTCGAG  ATTATCTTCGAAAGTCACGAGTTTTTG |
| --- | --- |
| shPHF19 #1-R | AATTCAAAAACTCGTGACTTTCGAAGATAAT  CTCGAGATTATCTTCGAAAGTCACGAG |
| shPHF19 #2-F | CCGGCCTCGTGACTTTCGAAGATAACTCGAG  TTATCTTCGAAAGTCACGAGGTTTTTG |
| shPHF19 #2-R | AATTCAAAAACCTCGTGACTTTCGAAGATAA  CTCGAGTTATCTTCGAAAGTCACGAGG |
| shSIAH1 #1-F | CCGGTGCATCAGCATAAGTCCATTACTCGAG  TAATGGACTTATGCTGATGCATTTTTG |
| shSIAH1 #1-R | AATTCAAAAATGCATCAGCATAAGTCCATTA  CTCGAGTAATGGACTTATGCTGATGCA |

Table 1

Table 2

| PHF19-F | ACTCGGGACTCCTATGGTGC |
| --- | --- |
| PHF19-R | CCTCCGTCAGTTTGGACATCA |
| β-catenin-F | CATCTACACAGTTTGATGCTGCT |
| β-catenin-R | GCAGTTTTGTCAGTTCAGGGA |
| SIAH1-F | AGCCGTCAGACTGCTACAG |
| SIAH1-R | AAAAGACTCGCCAAGTCATTGT |
| BTRC-F | ACCAACATGGGCACATAAACTC |
| BTRC-R | TGGCATCCAGGTATGACAGAAT |
| AXIN2-F | TACACTCCTTATTGGGCGATCA |
| AXIN2-R | TTGGCTACTCGTAAAGTTTTGGT |
| GSK3β-F | AGACGCTCCCTGTGATTTATGT |
| GSK3β-R | CCGATGGCAGATTCCAAAGG |
| FBXW7-F | GTCCCGAGAAGCGGTTTGATA |
| FBXW7-R | TGCTCAGGCACGTCAGAAAAG |

Table 3

| SAIH1-A-F | TTTCCATATCTGACTACATTTCTCCA |
| --- | --- |
| SAIH1-A-R | TCAGCCATTGCTTTACCACCT |
| SAIH1-B-1F | TCAACAACTGGTGGAGCCG |
| SAIH1-B-1R | ACTCTTCCAAACAGAACCAAAATG |
| SAIH1-C-1F | TTTTGGTTCTGTTTGGAAGAGTTT |
| SAIH1-C-1R | AGCCATCAGCCTGCCTTG |
| SAIH1-D-1F | GTTCGCACGATAAACCTGGAG |
| SAIH1-D-1R | GCTATTTTCGGTAGGTCAGAACAGT |
| SAIH1-E-1F | CTGAGGCTGTGAAACTTGAACG |
| SAIH1-E-1R | AAGAACAGCAGCCCAAATGAA |
